# Supplementary material for: Extracellular Cystatin F Is Internalised by Cytotoxic T Lymphocytes and Decreases Their Cytotoxicity
Source: Cancers (Basel). 2020 Dec 6;12(12):3660. doi: 10.3390/cancers12123660 (PMC7762138; doi:10.3390/cancers12123660)
Supplement: Supplementary file 1 [file cancers-12-03660-s001.pdf]

Supplementary Materials

Extracellular Cystatin F Is Internalised by Cytotoxic T Lymphocytes and Decreases Their Cytotoxicity

Mateja Prunk, Milica Perišić Nanut, Tanja Jakoš, Jerica Sabotič, Urban Švajger and Janko Kos

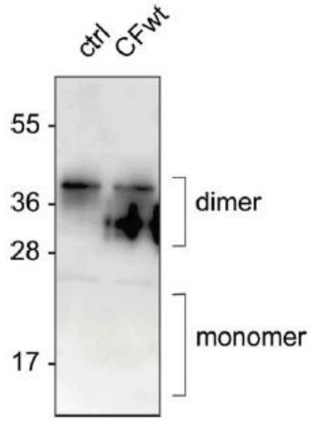

Figure S1. Extracellular full-length cystatin F is activated by N-terminal truncation after internalisation into TALL-104 cells. TALL-104 cells were either untreated (ctrl) or treated with 100 nM recombinant full-length cystatin F (CFwt) for 4 h. Activation after internalisation was assessed with cell lysates and western blot using an antibody directed against the N-terminal end of cystatin F. Since the antibody recognises the N-terminal part of cystatin F, it only detects the full-length form. The lack of monomeric band indicates that the internalised monomeric form of cystatin F (present on western blots performed with anti-cystatin F and anti-his-tag antibodies, Figure 1a,b) is N-terminally truncated.

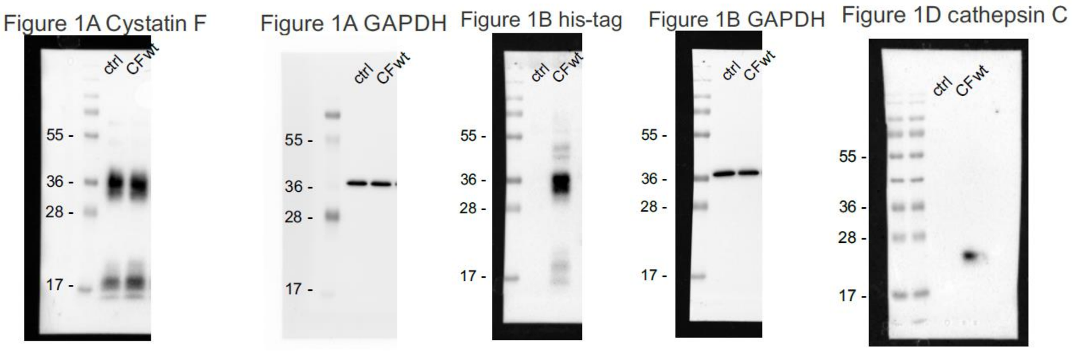

Detail information about Figure 1.

Figure 4A

Cystatin F b-actin

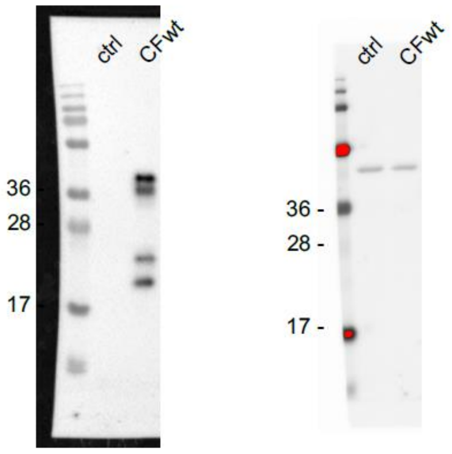

Figure 4B

cathepsin C b-actin

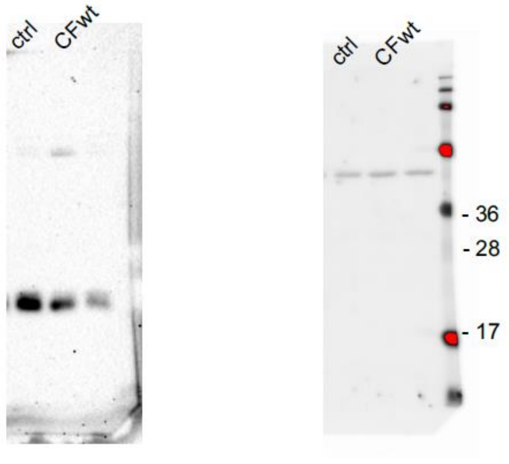

Figure 4D cathepsin C cathepsin H cathepsin L GAPDH

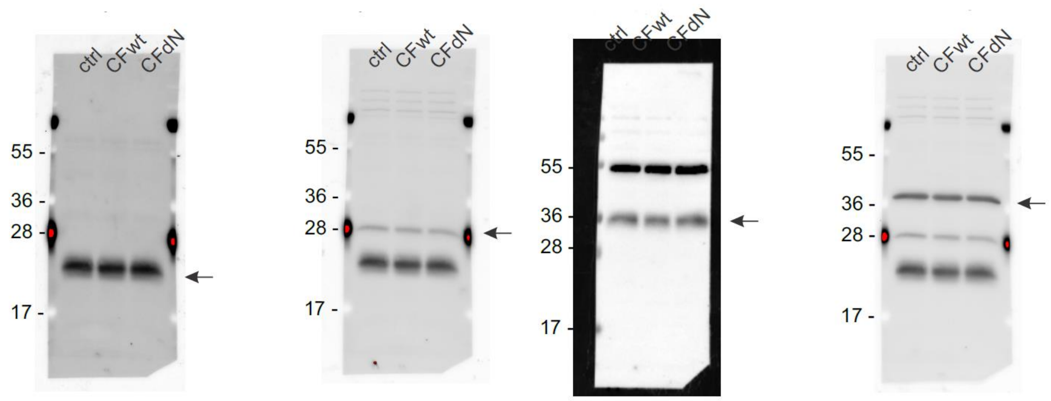

Figure 4E perforin

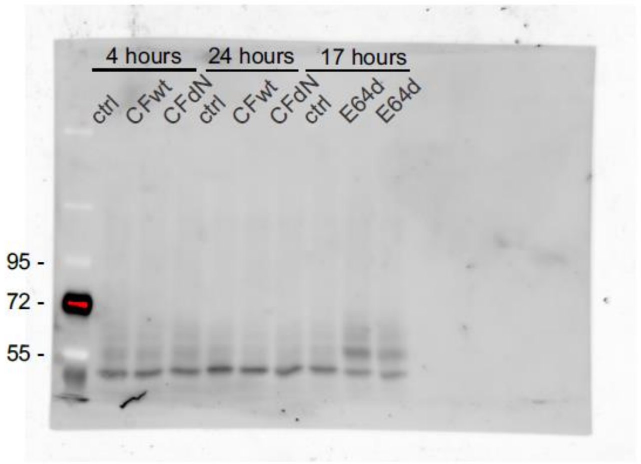

Detail information about Figure 4.

Figure 5A Cystatin F

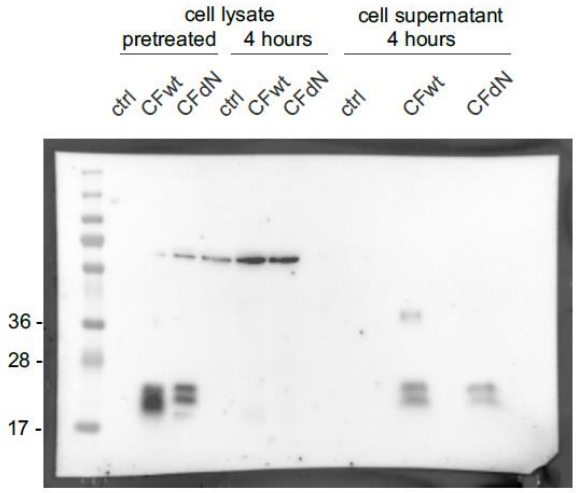

Figure 5B stain-free

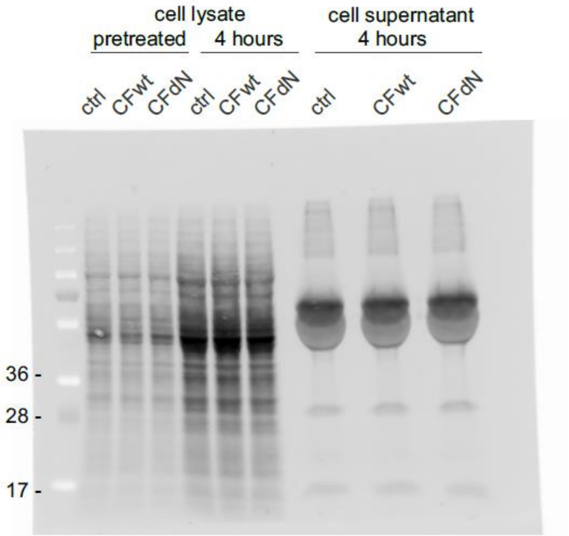

Detail information about Figure 5.

© 2020 by the authors. Licensee MDPI, Basel, Switzerland. This article is an open access article distributed under the terms and conditions of the Creative Commons Attribution (CC BY) license (http://creativecommons.org/licenses/by/4.0/).
